# Prediction of RNA-protein interactions using conjoint triad feature and chaos game representation

**DOI:** 10.1080/21655979.2018.1470721

**Published:** 2018-08-17

**Authors:** Hongchu Wang, Pengfei Wu

**Affiliations:** a Department of Mathematics, South China Normal University, Guangzhou P.R. of China; b College of Informatics, Huazhong Agricultural University, Wuhan P.R. of China

**Keywords:** RNA-protein interactions, conjoint triad feature, chaos game representation, random forest, prediction

## Abstract

RNA-protein interactions (RPIs) play a very important role in a wide range of post-transcriptional regulations, and identifying whether a given RNA-protein pair can form interactions or not is a vital prerequisite for dissecting the regulatory mechanisms of functional RNAs. Currently, expensive and time-consuming biological assays can only determine a very small portion of all RPIs, which calls for computational approaches to help biologists efficiently and correctly find candidate RPIs. Here, we integrated a successful computing algorithm, conjoint triad feature (CTF), and another method, chaos game representation (CGR), for representing RNA-protein pairs and by doing so developed a prediction model based on these representations and random forest (RF) classifiers. When testing two benchmark datasets, RPI369 and RPI2241, the combined method (CTF+CGR) showed some superiority compared with four existing tools. Especially on RPI2241, the CTF+CGR method improved prediction accuracy (ACC) from 0.91 (the best record of all published works) to 0.95. When independently testing a newly constructed dataset, RPI1449, which only contained experimentally validated RPIs released between 2014 and 2016, our method still showed some generalization capability with an ACC of 0.75. Accordingly, we believe that our hybrid CTF+CGR method will be an important tool for predicting RPIs in the future.

## Introduction

RNA-Protein Interactions (RPIs) play significant roles in various post-transcriptional regulation processes, such as RNA splicing, RNA transport, RNA replication, and mRNA translation [1–9]. A variety of functional RNAs, such as microRNAs (miRNAs), long non-coding RNAs (lncRNAs) and enhancer RNAs (eRNAs), usually work biologically through RNA-Protein Complexes (RPC) formed by the interactions between RNA binding proteins (RBPs) and these RNA macromolecules. Invalid interactions or mispairing could lead to human disease [10,11] or pathogen resistance in plants [12,13]. Therefore, determining whether a given RNA and a given RNA binding protein can form interactions or not is an essential prerequisite for dissection of RNA functions and regulatory mechanisms.

It is commonly believed that the best way to identify PRIs is to obtain the crystal structure of RPC by X-ray crystallography or Nuclear magnetic resonance (NMR) spectroscopy [14,15]. Nowadays, there are 1973 RPI complexes available in the Protein Data Bank (PDB, as of March 2017), which contains over 15,000 protein chains and more than 3,000 RNA chains. However, according to research using high-throughput sequencing techniques (such as RNA-Seq), at least 30,000 lncRNAs were identified by 2013 [16]. This number will increase rapidly every year, and specifically, studies have identified over 60,000 eRNAs in 2015 [17,18]. Obviously, the majority of those are not partnered with their target proteins (if they have partners), which calls for *in silico* prediction of RPIs.

With the rapid development of both high-throughput sequencing techniques and machine-learning algorithms, an increasing number of biological problems demand bioinformatic methods to achieve satisfactory solutions. However, in the area of RPI identifications, the research history is brief, and there are not many existing computational tools [19–28] because of the scarcity of available data.

The earliest work came from Pancaldi and Bähler in 2011 [19], who analysed the relationship between 40 RBPs and their target mRNA for 11 properties (more than 100 unique properties in total) and then trained support vector machine (SVM) and random forest (RF) classifiers using these properties to predict the interactions between RBPs and mRNA. In the same year, Bellucci et al. [20] developed a tool, called catRAPID, to give rapid predictions of RPIs by training on 592 RNA-protein pairs from the PDB. They used the physicochemical properties of sequences as features and found three most predictive features: secondary structure propensities, hydrogen bonding, and van der Waals [20]. Thereafter, Muppirala et al. [21] employed an idea from the Protein-Protein Interaction (PPI) prediction area, called Conjoint Triad Feature (CTF), to formulate protein sequences and then used normalized 4-gram frequencies to encode RNA sequences. They also constructed two benchmark datasets, called RPI369 and RPI2241, from PRIDB (a database of protein-RNA interfaces) [22] and achieved remarkable prediction accuracies by using CTF and 4-gram features on these two datasets. Two years later, Wang et al. [23] proposed a novel extended naive-Bayes-classifier to predict RPIs using the similar features of Muppirala et al. [21] Similar to catRAPID, Lu et al. [24] used the secondary structure, hydrogen-bonding, and the Van der Waals’ propensities as features and then employed matrix multiplication to give a score for each protein-lncRNA pairs obtained from the PDB database.

In 2015, Suresh et al. [25] integrated sequence information and predicted structure together to produce an accurate prediction of non-coding RNA-protein pairs on a newly-constructed dataset, called RPI1807. When tested on the RPI369 and RPI2241 datasets mentioned above, some improvements were achieved on prediction accuracies. Recently, Corrado et al. [26] developed a recommender system, named RNAcommender, to suggest candidate mRNA targets to the given RBPs by considering the domain information of proteins and predicted the structural information of RNA on datasets from the AURA 2, [27] which is a comprehensive database of experimentally determined interactions between transcription factor and human and mouse UTRs (untranslated regions in mRNAs). In 2016, Akbaripour-Elahabad et al. [28] integrated repetitive patterns and sequence motifs together with other traditional sequence composition features to predict RPIs, and the comparisons with other methods showed improvements on several of the datasets used previously.

Here, we propose a novel strategy by which integration of the successfully used CTF features and other important protein features, called chaos game representation (CGR), provides an accurate prediction of RPIs. To the best of our knowledge, there have been no reports in the area of RPI prediction that used the combination of CTF + CGR. CTF is a fundamental group of features to recognize the interaction of RNA and proteins and was shown to be successful in the majority of published prediction tools [21,23,25,28]. Furthermore, CGR is an important group of features for protein studies and achieved remarkable results in many prediction tools [29–33]. Detailed comparisons with existing tools using RPI369 and RPI2241 demonstrated that the combinations of these two features indeed got achieved improvements, suggesting that our prediction model will be an important tool for RPI prediction.

## Results

### Predicting rpis with CTF and CGR

In this study, we focused on how to use CTF + CGR methods for predicting RPIs. The first task was to transform the raw protein and RNA sequences into appropriate numerical vectors, which can represent intrinsic properties of their interactions. Here, we studied five different groups of representations of protein and RNA sequences and tried to determine which representation was optimal for predicting RPIs. After that, another important task was to choose a powerful machine-learning algorithm or a classifier to discriminate true RPIs and non-RPIs based on the above representations. We employed random forest as our classifier, which had been proven as a successful tool for predicting RPIs [21,24,28]. Additionally, the 10-fold cross validation test was adopted for testing the prediction ability of five different models.

For the first model, we used the fundamental feature set, Amino Acid Composition (AAC, 20- dimension), for protein combined with Nucleotide Composition (NC, 4-dimension) for RNA as the background for comparisons. Then, four feature sets (CTF, CGR, CTF + CGR and CTF + CGR + AAC + NC) were run with RF to show the prediction results. Note that for CTF, the feature set contains 343 features of *CTF*_protein_ and 256 features of *CTF*_RNA_ (for details of these 256 features, please see the subsection ‘Features of RNAs for prediction-Conjoint triad feature’), which leads to 599 features (i.e., 343 + 256) in the total CTF feature set. Similarly, for CGR, the feature set contains 24 features of *CGR*_protein_ and 16 features of *CG*R_RNA_ (for details of these 16 features, please see the subsection ‘Features of RNAs for prediction- Chaos game representation’), which counts 40 features (i.e., 24 + 16) of CGR feature set. Finally, CTF + CGR feature set simply takes the CTF feature set and the CGR feature set together to form a combined feature set which contains 639 features (599 + 40) in total, and CTF + CGR + AAC + NC feature set takes the first three feature set together, which leads to 663 features (599 + 40 + 20 + 4) in all. Importantly, we used RF classifier separately on each feature representation as the training matrix and evaluated the corresponding predicting performance of each feature representation.

We listed the detailed prediction results for RPI369 dataset in Table 1, from which the fundamental feature set AAC + NC clearly demonstrated the highest prediction accuracy (ACC) with 0.6965. The feature set CTF produced a satisfactory result with ACC of 0.7954, which performed much better than AAC + NC. Interestingly, the prediction accuracy increases to 0.7995 with the combination of CTF + CGR, which implies that CTF and CGR are two complementary feature sets.

**Table 1.** Results in predicting RPIs on RPI369 dataset (10-fold cross-validation test).

Feature set	Dim	Sens	Spec	ACC	MCC	AUC	ntree	mtry
AAC+NC	20 + 4 = 24	0.6856	0.7073	0.6965	0.3930	0.7011	372	17
CTF	343 + 256 = 599	0.8211	0.7696	0.7954	0.5916	0.8295	487	476
CGR	24 + 16 = 40	0.7019	0.7317	0.7168	0.4338	0.7559	338	15
CTF+CGR	599 + 40 = 639	0.8211	0.7778	0.7995	0.5995	0.7842	489	442
CTF+CGR +AAC+NC	599 + 40 + 20 + 4 = 663	0.7466	0.8010	0.7724	0.5500	0.8198	327	142

Similarly, we listed the prediction results on RPI2241 dataset in Table 2, from which we could find analogous patterns: the prediction accuracy of AAC + NC was lowest (ACC = 0.8134), and CTF + CGR + AAC + NC achieved better ACC of 0.8536. The encouraging result of CTF + CGR showed that the combination got significant improvement with ACC of 0.9520, which further confirmed that CTF and CGR were a powerful combinatory feature set for RPI prediction.

**Table 2.** Results in predicting RPIs on RPI2241 dataset (10-fold cross-validation test).

Feature set	Dim	Sens	Spec	ACC	MCC	AUC	ntree	mtry
AAC+NC	20 + 4 = 24	0.7964	0.8298	0.8134	0.6268	0.8791	437	10
CTF	343 + 256 = 599	0.8415	0.8568	0.8492	0.6984	0.9163	426	406
CGR	24 + 16 = 40	0.7964	0.8659	0.8316	0.6643	0.8867	422	9
CTF+CGR	599 + 40 = 639	0.9192	0.9848	0.9520	0.9060	0.9722	482	104
CTF+CGR +AAC+NC	599 + 40 + 20 + 4 = 663	0.8405	0.8667	0.8536	0.7073	0.9163	385	306

**Figure 1.**
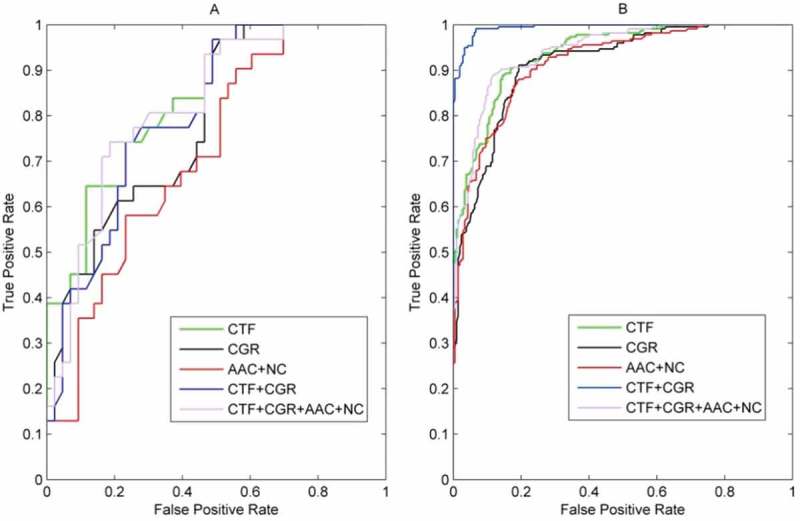
ROC curves of five groups of features on RPI369 (A) and RPI2241 (B).

We generated the ROC curves for the five models on RPI369 (Figure 1(a)) and RPI2241 (Figure 1(b)). The resulting AUC values showed some interesting results: on RPI369 dataset, the ACC value of a combination of CTF + CGR was optimal with 0.7995, but the AUC value was only 0.7842, which was smaller than that of CTF (0.8295). When turning to RPI2241 dataset, the AUC value of a combination of CTF + CGR achieved 0.9722, which was much larger than that of CTF (0.9163). The conclusion remains consistent comparing either AUC or ACC value, and the relatively low value of the combination of CTF + CGR can be explained by fewer samples in the RPI369 dataset.

The two parameters (ntree and mtry) in the RF models vary significantly between the different datasets. For example, the value for mtry (the number of input variables randomly chosen at each split) is 476 for CTF, 15 for CGR, and 442 for the combination. Recall that the total dimensions of CTF, CGR and the combination are 599, 40 and 639, respectively; the value of 476 of mtry in the model CTF implies that 476 important features among the total 599 features are selected as the optimal feature set to reach the best prediction result. RF selects 476 optimal features among all the 599 features, which means the unselected features are substitutable and the reasons might be the high correlations between the selected features. Interestingly, the value of mtry falls to 442 and does not equal 491 (476 + 15) when we combine CTF and CGR (the total number of features reaches to 639). Note that the values of mtry of CTF and CGR are 476 and 15 respectively, which means RF selects 476 significant features from CTF and selects 15 ones from CGR. Intuitively, one expect the value of mtry of the combination will be 491 (476 + 15), but the exact value of mtry is 442 that is much smaller. The reason might be the correlations between 476 selected features of CTF and 15 selected features of CGR, which makes RF select representative 442 features of the combination.Together with the best ACC of 0.7995 among all the feature sets, this result implies that CTF and CGR are truly complementary feature sets and that the combination further compresses the redundant information to reach the best prediction result.

### Comparisons with existing methods

To show the superiority of our method, comprehensive comparisons with four existing tools (Muppirala et al [21]., Wang et al [23]., RPI-Pred [25], rpiCOOL [28]) were listed in Table 3. Among existing prediction methods, RPI-Pred [25] performed best of those tested on the RPI369 dataset, and rpiCOOL [28] performed better than others on the RPI2241dataset. Table 3 shows that our method achieved the second rank when testing on RPI369, and encouragingly, our method ranked first when testing on RPI2241. As RPI369 only contained 369 RNA-protein interaction pairs, the models developed on this small sample size will not guarantee generalization capability. In contrast, models developed on RPI2241 used more training samples and will be more reliable for prediction on blind samples (the samples with no experiment information). On this point, because our method achieved the best prediction accuracy of 0.95 on RPI2241, we believe that our method outperforms the four existing prediction tools.

**Table 3.** Comparisons with four existing tools.

Tools	RPI369			RPI2241		
	ACC	MCC	AUC	ACC	MCC	AUC
Muppirala et al. [21]	0.76	–	–	0.90	–	–
Wang et al. [23]	0.77	0.46	–	0.76	0.42	–
RPI-Pred [25]	0.92	–	0.95	0.84	–	0.89
rpiCOOL [28]	0.80	0.60	0.88	0.91	0.81	0.97
Our method	0.80	0.60	0.78	0.95	0.91	0.97

## Discussion

To test the generalization ability of our model, we constructed a new dataset, named RPI1449, to test our model independently. Similar to previous strategies [24,25], we searched the PDB database (http://www.pdb.org) for complexes that only contains protein chains and RNA chains; 1973 protein-RNA complexes were displayed during the search results. To avoid overlaps between RPI369 and RPI2241, we chose only a subset of those complexes that were reported from 1 January 2014 to 31 December 2016. This way, 849 complexes were selected and advanced to the next step. To achieve statistical significance, we removed pairs of protein and RNA chains that were simultaneously shorter than 25 amino acids and 10 bases, respectively. To correctly select RNA-protein pairs that have real interactions, we employed the same strategy as Suresh et al [25]. to confirm that a given protein chain and RNA chain had physical interactions by identifying at least two atoms, one from protein and another from RNA, with an intermolecular distance less than 3.4 Å. From the above three criteria, 1449 RNA-protein pairs (in dataset RPI1449) were considered as the independent test dataset (see Table 4).

**Table 4.** Independent testing dataset and predicting result.

Data sources	RNA-protein complexes in PDB database	Independent testing dataset RPI1449	Comparisons of predicting results	
			Muppirala et al. [21]	Our test result
2014	378	1449 RNA-protein pairs after preprocessing	ACC: 1042/1449 = 0.7191	ACC: 1092/1449 = 0.7536
2015	221			
2016	250			
Total	849			

For a blind independent test, all the 1449 newly built RNA-protein pairs were put into the prediction model, which was previously developed based on the RPI2241 dataset, and then recorded the corresponding prediction accuracy. Note that RPI2241 was built in 2011, while 1449 RNA-protein pairs were constructed based on the RNA-protein complexes that were released between 2014 and 2016 in the PDB database. Therefore, no overlap exists between these two datasets, and the experiment is called the blind independent test. We compared our prediction result with the method of Muppirala et al [21]. on the independent test using the measurement of accuracy. The comparison results are shown in Table 4, which indicate that 1092 out of 1449 RNA-protein pairs were correctly predicted in the independent test, leading to a predicting accuracy of 0.7536. This value is about 3% higher than the accuracy rate produced using the method of Muppirala et al., which implies that our prediction model has generalization capability.

## Materials and methods

### Datasets

In this paper, we used two datasets for training and testing. Now, RPI369 and RPI2241 are two famous benchmark datasets that were used in many previous studies [23–25,28] for comparison. Here, we also use RPI369 and RPI2241 for training and testing our method. To download RPI369 and RPI2241 or inquire about detailed information, one can refer to Muppirala et al [21].

### Features of proteins for prediction

For feature extraction of protein sequences, each protein chain was formulated as a numerical vector that would be input into RF for classification. Here, we employed the following two methods for representing the protein chains:

1. Conjoint triad feature

Conjoint triad feature (CTF) was a successful method for PPI prediction for its powerful ability to detect interaction interfaces [34] and was first applied to predict RPI by Muppirala et al [21]., which produced some satisfactory results. It is noteworthy that almost all the subsequent studies used CTF as sequence features, or at least a part of features [23–25,28], and it has since become a dominant method in RPI prediction.

**Figure 2.**
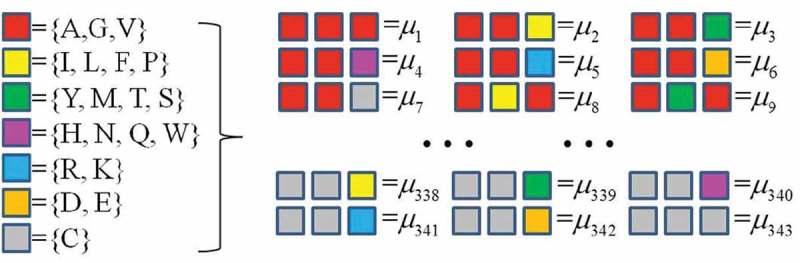
CTF picture of protein.

Specifically, CTF divides all 20 amino acids into seven groups ({AGV}, {ILFP}, {YMTS}, {HNQW}, {RK},{DE}, {C}) according to their physicochemical properties and then considers all the amino acids in the same group as identical. Then, CTF considers all sets of three successive amino acids (triad) within a given protein sequence and counts the triad frequencies by computing the occurrence numbers of all 343 triads (7 × 7 × 7) (Figure 2).

Mathematically, we denote a protein sequence *P* with length *L* as

(1) $$P=P_{1}P_{2}P_{3}\cdots P_{L}.$$

Then, we consider all the successive three amino acids in *P*, that is $P_{1}P_{2}P_{3}$,$P_{2}P_{3}P_{4}$,$\cdots P_{3}P_{4}P_{5}$, …, $P_{L-2}P_{L-1}P_{L}$, The *CTF*_protein_ is defined as the normalized frequency of each triad in *P*, i.e.,

(2) $$CTF_{protein}=[f_{1},f_{2},f_{3},\cdots \;,f_{343}{]}^{T}$$

where $f_{i}=\frac{m_{i}}{L-2}$, and $m_{i}\;$is the occurrence number of the *i*-th triad $\mu_{i}\;$with each i(i=1,2,⋯,343). As a result, *CTF*_protein_ encodes each protein sequence into a 343-dimensional numerical vector.

2. Chaos game representation

Chaos game representation (CGR) is another important method to formulate protein sequence and was also successfully used in many protein studies [29–33]. It originally applied the idea of Iterated Function System (IFS) from the fractal theory for generating CGR picture of DNA sequence in 1990 [35], and then was employed to generate CGR picture of protein sequence in 1997 [36]. Thereafter, several research studies have focused on how to extract useful features from CGR picture and showed that those extracted features played important roles in some protein studies [29–33]. Here, we adopt one group of the used features, called CGR-24, to formulate protein sequences [31–33].

**Figure 3.**
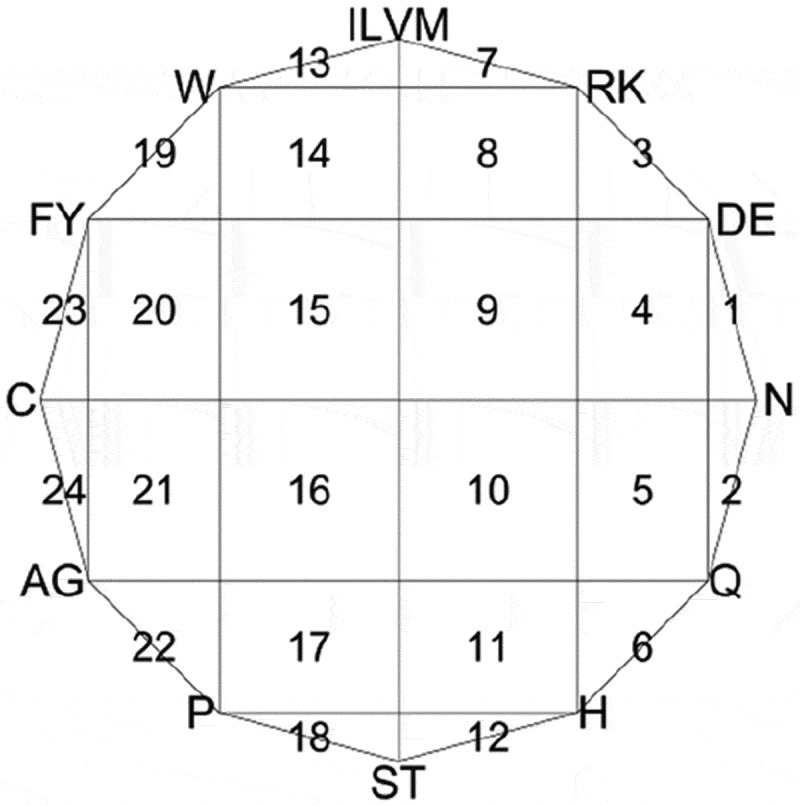
CGR picture of protein. The segments labelled serially with numbers 1-24.

More precisely, we first draw a 12-sided regular polygon with each vertex representing a specific group of amino acids (Figure 3). Then, we set the centre of polygon as the initial point, and when we read an alphabet from a given protein sequence with length L in order, a new point was drawn half way between the initial point and the vertex labelled by this alphabet. Next, we set the point just drawn to be the new initial point, and subsequently, L points can be drawn with such iteration.

Mathematically, the coordinates of all 12 vertexes ($V_{k}(x,y),\;k=1,2,\cdots 12$) can be computed as ($V_{1}(1,0)$ as the first vertex of the polygon):

$$\left\{\begin{array}{c} V_{k}(x)=\cos\frac{k-1}{6}\pi \\ V_{k}(y)=\sin\frac{k-1}{6}\pi \end{array},\;k=2,3,\cdots \;12.\right.$$

The coordinates of L successively-drawn points ($CGR_{i}(x,y),\;i=1,2,\cdots ,L$) can be given by:

$$\left\{\begin{array}{c} CGR_{i}(x)=\frac{1}{2}(CGR_{i-1}(x)+V_{i}(x))\\ CGR_{i}(y)=\frac{1}{2}(CGR_{i-1}(y)+V_{i}(y)) \end{array},\;\;i=1,2,3,\cdots \;L.\right.$$

Finally, we divide the whole polygon into 24 segments that are labelled in Figure 3, and CGR-24 counts the point frequencies of all 24 segments. Under this procedure, CGR-24 transforms a given protein sequence into a 24-dimensional numerical vector. For more detailed information of CGR-24 features, one can refer to some previous studies [31–33].

### Features of RNA for prediction

In this section, we describe how to formulate RNA chains by *CTF*_RNA_ and CGR-16 methods.

1. Conjoint triad feature

**Figure 4.**
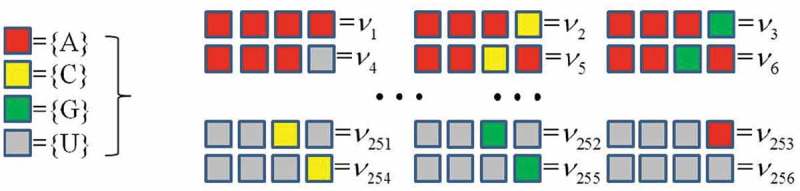
CTF picture of RNA.

Similar to the algorithm of protein, CTF of a RNA chain considers all the successive four bases in a given RNA sequence and counts the occurrence frequencies of all 4 × 4 × 4 × 4 = 256 4-mer types (Figure 4). Mathematically, we denote a RNA sequence ω with *N* bases as

(1) $$\omega =R_{1}R_{2}R_{3}\cdots \;R_{N}.$$

Then, we consider all the successive four bases in ω, that is $R_{1}R_{2}R_{3}R_{4}$,$R_{2}R_{3}R_{4}R_{5}$,$\cdots ,R_{N-3}R_{N-2}R_{N-1}R_{N}$. The *CTF*_RNA_ features is defined as the normalized frequency of each 4-mer in ω, i.e.,

(2) $$CTF_{RNA}=[f_{1},f_{2},f_{3},\cdots \;,f_{256}{]}^{T},$$

where $f_{i}=\frac{n_{i}}{N-2}$, and $n_{i}$ is the occurrence number of the *i*-th 4-mer $\nu_{i}$ with each i(i=1,2,⋯,256). This way, *CTF*_RNA_ encodes each RNA sequence into a 256-dimensional numerical vector.

2. Chaos game representation

**Figure 5.**
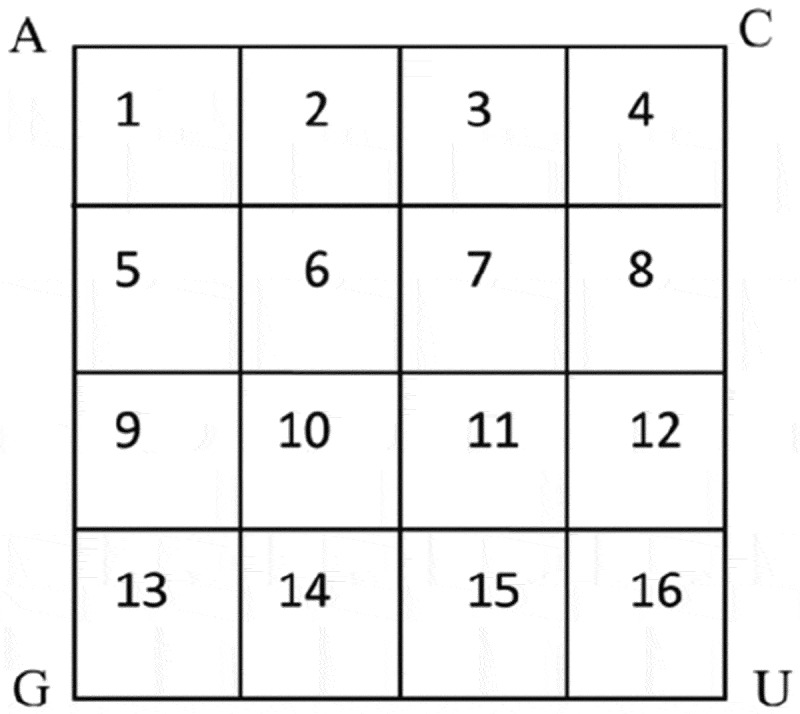
CGR picture of RNA. The segments labelled serially with numbers 1-16.

The drawing algorithm of CGR picture of RNA is almost the same as that of protein, and the only difference is that the 12-sided regular polygon is replaced by a square with four vertexes representing A, C, G, U (Figure 5).

Mathematically, the coordinates of four vertexes are denoted as $V_{1}(0,0)$,$V_{2}(1,0)$,$V_{3}(1,1)$,$V_{4}(0,1)$, and the coordinates of successively-drawn points can be given by:

$$\left\{\begin{array}{c} CGR_{i}(x)=\frac{1}{2}(CGR_{i-1}(x)+V_{i}(x))\\ CGR_{i}(y)=\frac{1}{2}(CGR_{i-1}(y)+V_{i}(y)) \end{array},\;\;i=1,2,3,\cdots \;N.\right.$$

Finally, we divide the whole square into 16 segments, as is shown in Figure 5, and then CGR-16 counts the occurrence frequencies of all 16 segments. More precisely, all 16 segments are denoted by$\;S_{k},\;k=1,2,\cdots \;,16$, and also denote $L_{k},k=1,2,\cdots \;,16$ to be the number of points that fall into $S_{k}$. Then set

(3) $$D_{k}=\frac{L_{k}}{N},k=1,2,\cdots \;,16,$$

to be the occurrence frequencies of all 16 segments. This way, CGR-16 encodes each RNA sequence as a 16-dimensional vector$\left(D_{1},D_{2},\cdots ,D_{16}\right)$.

### Random forest

Random forest (RF) is a popular machine-learning method for classification or regression tasks. There are two advantages of RF: 1) easy training that requires researchers to tune only two internal parameters, ‘ntree and mtry’ during the training approach, and 2) powerful prediction ability on various datasets when comparing other machine-learning or statistical methods. These properties have helped make RF become one of the most successful machine-learning tools in the last two decades. Actually, it is an ensemble machine learning method whose prediction result is voted by a certain number of decision trees. Each tree is independently constructed with a bootstrap sample of the training set. Additionally, comprehensive theory and wide applications of RF can be found in a famous paper written by Breiman [37]. Here, we adopt a MATLAB toolbox of RF, which is available at http://code.google.com/p/randomforest-matlab/, to train and test our model. We chose the optimal combination of the two parameters of ‘ntree’ in [300,500] and mtry in [1,*n*] (*n* is the number of the total features in that dataset) and adopted the grid optimization to find the globe optimal solution.

### Evaluation of the prediction performance

For evaluating the predicting performance, we adopted 10-fold cross-validation [38] to examine its’ effectiveness. Additionally, performance of our predictor is quantitatively measured by the following common-used indexes: sensitivity (**Sens**), specificity (**Spec**), accuracy (**ACC**) and Matthew’s correlation coefficient (**MCC**) value, which are calculated as:

(11) $$\left\{\begin{array}{c} Sens=\frac{TP}{TP+FN}\\ Spec=\frac{TN}{TN+FP}\\ ACC=\frac{TP+TN}{TP+FP+TN+FN}\\ MCC=\frac{TP\times TN-FP\times FN}{\sqrt{\left(TP+FN)(TP+FP)(TN+FP)(TN+FN\right)}} \end{array}\right.$$

Importantly, the ROC curves and the corresponding area under the curve (AUC) is another important index for testing the balance between true positive and false positive rates. In total, we used five indexes (**Sens, Spec, ACC, MCC, AUC**) for comprehensively measuring the predicting performance of a given predictor (see Tables 1 and 2).

## Conclusion

In this paper, we integrated CTF and CGR to give appropriate representations of RNA-protein interaction pairs and developed a prediction model of RPIs based on these representations and random forest. A number of previous studies all used CTF as representations of RNA-protein pairs and achieved remarkable prediction performances [21,23,25]; CTF was considered the most important feature for RPI prediction. Up to this point, our work has shown that prediction performance can be significantly improved by adding CGR representations, which is the most significant finding of our current study.

For detailed information of prediction results, when training and cross validating two benchmark datasets, RPI369 and RPI2241, the combined representation of CTF + CGR achieved the best prediction performance. Compared with four existing tools [21,23,25,28], the prediction model constructed from the combinatorial features of CTF + CGR showed some improvements, especially on RPI2241. Furthermore, a new independent testing dataset, RPI1449, was built using new experimentally validated RNA-protein interactions, and a blind independent test was performed. The corresponding prediction accuracy of 0.7536 demonstrated that our method has some generalization capability. In conclusion, the combinational representation of CTF + CGR appears to be a powerful method for RPI prediction, and our model based on CTF + CGR and random forest may prove to be an important tool for prediction of RPIs.

## Acronyms

• RNA-protein interactions (RPIs)

• prediction accuracy (ACC)

• RNA-Protein Complex (RPC)

• Conjoint Triad Feature (CTF)

• Chaos Game representation (CGR)

• RNA-Protein Complex (RPC)

• RNA Binding Proteins (RBPs)

• long non-coding RNA (lncRNA)

• microRNA (miRNA)

• enhancer RNA (eRNA)

• Amino Acid Composition (AAC, 20-dimension)

• Nucleotide Composition (NC, 4-dimension)

• untranslated regions in mRNAs (UTRs)

• Nuclear magnetic resonance (NMR)
